# New Insight Into Neutrophils: A Potential Therapeutic Target for Cerebral Ischemia

**DOI:** 10.3389/fimmu.2021.692061

**Published:** 2021-07-14

**Authors:** Ran Chen, Xu Zhang, Lijuan Gu, Hua Zhu, Yi Zhong, Yingze Ye, Xiaoxing Xiong, Zhihong Jian

**Affiliations:** ^1^ Department of Neurosurgery, Renmin Hospital of Wuhan University, Wuhan, China; ^2^ Central Laboratory, Renmin Hospital of Wuhan University, Wuhan, China

**Keywords:** stroke, neuroinflammation, ischemia, neutrophils, blood-brain barrier, NETs, ROS

## Abstract

Ischemic stroke is one of the main issues threatening human health worldwide, and it is also the main cause of permanent disability in adults. Energy consumption and hypoxia after ischemic stroke leads to the death of nerve cells, activate resident glial cells, and promote the infiltration of peripheral immune cells into the brain, resulting in various immune-mediated effects and even contradictory effects. Immune cell infiltration can mediate neuronal apoptosis and aggravate ischemic injury, but it can also promote neuronal repair, differentiation and regeneration. The central nervous system (CNS), which is one of the most important immune privileged parts of the human body, is separated from the peripheral immune system by the blood-brain barrier (BBB). Under physiological conditions, the infiltration of peripheral immune cells into the CNS is controlled by the BBB and regulated by the interaction between immune cells and vascular endothelial cells. As the immune response plays a key role in regulating the development of ischemic injury, neutrophils have been proven to be involved in many inflammatory diseases, especially acute ischemic stroke (AIS). However, neutrophils may play a dual role in the CNS. Neutrophils are the first group of immune cells to enter the brain from the periphery after ischemic stroke, and their exact role in cerebral ischemia remains to be further explored. Elucidating the characteristics of immune cells and their role in the regulation of the inflammatory response may lead to the identification of new potential therapeutic strategies. Thus, this review will specifically discuss the role of neutrophils in ischemic stroke from production to functional differentiation, emphasizing promising targeted interventions, which may promote the development of ischemic stroke treatments in the future.

## Introduction

Inflammation plays a dual role in the pathogenesis and prognosis of central nervous system (CNS) injury, exerting both beneficial and harmful effects (1). After stroke, damaged brain tissue causes systemic inflammation, including the recruitment of immune cells. This response is mediated, at least in part, by sympathetic activation of the bone marrow. Neutrophils are the first immune cells to accumulate in ischemic brain regions, which occurs within a few minutes after injury (2). Recent studies have shown that immune elements are closely involved in all stages of the ischemic cascade, from acute ischemic events caused by blood supply interruption to brain parenchymal injury and subsequent tissue repair. Conversely, the ischemic brain can also exert strong inhibitory effects on lymphoid organs through the autonomic nervous system, thereby promoting the occurrence of complications, which are the main determinants of the incidence and mortality of stroke (3). Therefore, the immune system is closely related to the long-term prognosis and survival of patients with ischemic stroke. After stroke, the acute interruption of blood supply causes primary irreversible tissue damage, leading to nerve cell death, which occurs in the ischemic core; nerve cell death leads to the subsequent release of damage-associated molecular patterns (DAMPs). Subsequent brain damage to the periinfarct area (ischemic penumbra) is caused by a series of secondary events, such as excitotoxicity, oxidative stress and mitochondrial dysfunction. Neutrophils are traditionally considered the first line of defense for innate immunity (4), and have been shown to be involved in many inflammatory diseases, especially acute ischemic stroke (AIS). However, neutrophils may play a dual role in the central nervous system after ischemic stroke. The activation of neutrophils leads to the release of nuclear and particulate matter, forming a wide network of DNA complexes (neutrophil extracellular traps, NETs) (5). TLRs can also detect endogenous molecules called damps, which are produced by tissues or immune cells in response to injury or infection. TLR activation of neutrophils leads to important cellular processes, including the production of reactive oxygen species (ROS) and cytokines, all of which may be involved in the pathogenesis of chronic inflammation when inflammation and immune imbalance. In turn, inflammation and tissue damage lead to the release of endogenous TLR ligands, known as damage associated molecular patterns (DAMPs), which are a rapidly growing class of potent inflammatory stimuli and widely released in inflammatory centers after ischemic stroke (6). Part of the specific mechanism can be reflected by the active or passive release of nuclear protein, high-mobility group protein B1 (HMGB1), which activates TLR2/4 on the surface of neutrophils, leading to NADPH oxidase assembly and ROS generation, up regulating the production of anti-apoptotic protein Bcl-xl and pro-inflammatory cytokines, thus aggravating the process of inflammatory response (7). Phagocytosis triggers the activation of neutrophils, resulting in the release of antimicrobial peptides, proteases, myeloperoxidase (MPO) and superoxide anion $\left(O_{2}^{-}\right)$, which produce reactive oxygen species (ROS) *via* a series of cascade reactions (8). At sites of inflammation, high levels of proinflammatory cytokines such as GM-CSF and tumor necrosis factor (TNF)a can enhance the release of ROS. Excessive activation of NADPH oxidase in the neutrophil cascade leads to excessive production of ROS. ROS can activate the production of matrix metalloproteinase-9 (MMP-9) through a signaling pathway and then lead to tissue damage and excessive inflammatory reactions (9), such as microvascular injury and blood-brain barrier (BBB) damage then cause the entry of more peripheral neutrophils into the damaged area, aggravating the response to nerve injury (5). However, neutrophils also have beneficial effects, as they participate in the core pathophysiological process of neutrophil-driven repair, namely, angiogenesis, which is an important process by which nutrients and oxygen are transported to healing tissue. Endothelial progenitor cells recruited by activating N-formyl peptide receptor 2 (FPR2) promote reendothelialization by directly covering the injured site but also initiate vascular repair *via* vascular endothelial growth factor (VEGF) and epidermal growth factor (EGF) in a paracrine manner (10, 11). Here, we reassess the contribution of inflammation and immunity to the pathophysiology of stroke. In this review, we will focus on the spatiotemporal role of neutrophils in ischemic brain injury, evaluate their effects on tissue damage and repair, and further explore the possible molecular and immune mechanisms underlying these effects. In addition, we propose a new possible therapeutic strategy involving local targeting of neutrophils at the onset of cerebral ischemia rather than nonselective overall inhibition of neutrophil function. 

## The Role of Neutrophils in Ischemic Brain Injury

**Figure 1:**
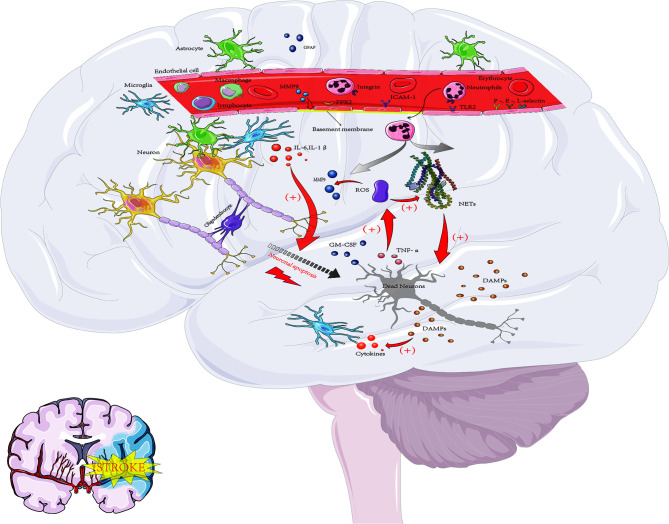
The inflammatory response after cerebral ischemia. Acute ischemic events lead to oxidative stress and excitotoxicity, which lead to the activation of microglia and astrocytes and then promote the secretion of cytokines, MMPs and GFAP(glial fibrillary acidic protein). These proinflammatory signaling molecules lead to upregulation of the expression of ICAM-1 and selectins on endothelial cells and promote the entry of neutrophils, macrophages, lymphocytes and other blood-derived inflammatory cells into the ischemic area. Neutrophils are the first group of leukocytes to enter the CNS. At inflammatory sites, high levels of proinflammatory cytokines such as GM-CSF and TNF-α can enhance the release of ROS. ROS can induce the production of MMP-9 through signaling pathways, which leads to tissue damage and excessive inflammatory responses, such as microvascular basement membrane damage and BBB damage, and then cause more peripheral neutrophils to enter the damaged area, aggravating the nerve injury response. The activation of neutrophils can also lead to the release of nuclear and particulate matter, which form a wide network of DNA complexes (NETs) that further aggravate neuronal damage. In addition, DAMPs, which in turn activate microglia and peripheral immune cells (neutrophils, macrophages and lymphocytes), are released by dying neurons, resulting in the production of proinflammatory factors and thus leading to further activation of neutrophils. These pathological events lead to neuronal death and further increase damage to the ischemic brain.

**Figure 2:**
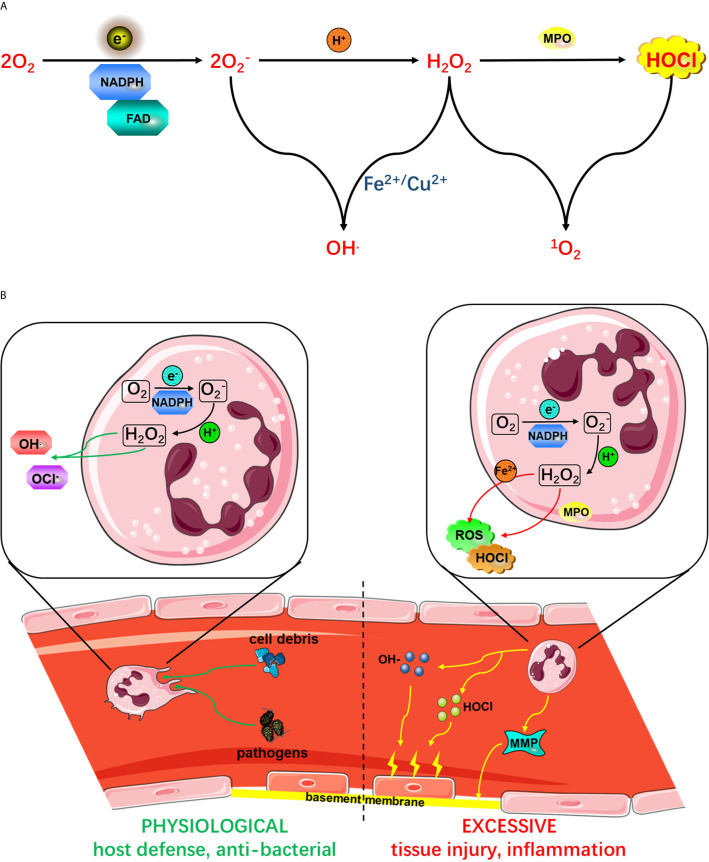
**(A)** Active NADPH oxidase complexes promote ROS production. The activated NADPH oxidase complex uses fad to transfer electrons (e^-^) to oxygen to form $O_{2}^{-}$ from phagocytes. $O_{2}^{-}$ is produced by the activation of NADPH oxidase. $O_{2}^{-}$. $O_{2}^{-}$ from phagocytes. $O_{2}^{-}$ is produced by the activation of NADPH oxidase. $O_{2}^{-}$ produced by NADPH oxidase can react with protons to form H_2_O_2_, which in turn produces HOCl under the action of MPO. $O_{2}^{-}$ from phagocytes. $O_{2}^{-}$ is produced by the activation of NADPH oxidase. $O_{2}^{-}$ can react with H_2_O_2_ in the presence of Fe^2+^ or Cu^2+^ to form OH- or ROS. **(B)** The role of neutrophil activation in host defense and the inflammatory response. As shown on the left side, the initiation of proinflammatory cytokines or microbial molecules under physiological conditions is an immune-monitoring mechanism that ultimately enhances the antibacterial activity of neutrophils. However, as shown on the right side, excessive activation of neutrophil NADPH oxidase leads to excessive production of ROS, causing tissue damage and an excessive inflammatory response. Mediated by various proinflammatory mediators, receptor signals on the vascular endothelial surface are involved in neutrophil rolling, adhesion and endothelial barrier crossing. Phagocytosis of neutrophils leads to the activation of a series of processes, leading to the release of antimicrobial peptides, proteases, MPO and $O_{2}^{-}$ from phagocytes. $O_{2}^{-}$ is produced by the activation of NADPH oxidase. $O_{2}^{-}$, which is produced by the activation of NADPH oxidase, from phagocytes.

Considering their central role in early brain accumulation and subsequent immune responses, neutrophils may be key targets for brain injury and stroke recovery. Neutrophils are the first blood-derived immune cells to invade ischemic tissue, followed by monocytes (12). After stroke, neutrophils migrate through the endothelial vessel wall. Later, neutrophils are attracted to the ischemic area along a concentration gradient of chemokines. Neutrophils further cause secondary injury by releasing proinflammatory factors, ROS, proteases and matrix metalloproteinases (MMPs) (13) (**Figures 1** and **2**). These factors damage the endothelial cell membrane and basal layer, leading to changes in BBB permeability and edema after ischemia. After neutrophil invasion (4-6 hours), monocytes adhere to the vascular wall and move into the ischemic area. The activity of monocytes peaks 3-7 days after cerebral ischemia (14). Increasing evidence from animal models of ischemic stroke and cerebral hemorrhage supports this view. A model ischemic stroke induced by intracavitary middle cerebral artery occlusion (MCAO) was reported in 1994, and it was found that neutrophils accumulated in cerebral vessels and veins as early as 30 minutes after MCAO. Gelderblom et al. used immunohistochemistry and flow cytometry to quantify the infiltrated immune cell subsets. They analyzed the time dynamics of immune cell accumulation in rodent stroke models in detail. Circulating monocytes and macrophages are first detected in capillaries and venules, and the numbers of these cells in the brain increase from 4 to 6 hours after stroke, peaking on days 2 to 7 (15). Neutrophils play a major role in acute ischemic injury and may also lead to atherosclerosis and thrombosis (16).

To clearly assess the potential accumulation of neutrophils, Enzmann et al. studied the temporal and spatial localization of PMNs after transient middle cerebral artery occlusion (t MCAO) in mice and human stroke samples (17). Using specific markers that can specifically label PMN (Ly6G) and monocyte/macrophage (Ly6C) and defining the cell and basement membrane boundaries of neurovascular unit (NVU), histology and confocal microscopy showed that almost no PMN entered the infarcted CNS parenchyma. No matter the duration of t MCAO, PMNs are mainly confined to the lumen surface or perivascular space. Their results showed that neurovascular units rather than brain parenchyma were the main parts of PMN after central nervous system ischemia (17). Using *in vivo* two-photon microscopy combined with conventional immunohistochemistry, researchers subsequently demonstrated that neutrophils rapidly attach to inflammatory brain endothelial cells in mouse models of permanent peripheral and transient intracavitary MCAO (18). After this attachment, the cells migrate to the brain parenchyma. Invasive neutrophils interact with local microglia rapidly and are engulfed by them (19); this was previously observed in an ischemic tissue model *in vitro* (20). Another similar study recently confirmed this observation in a more advanced animal model in which microglia and neutrophils express highly specific and unique fluorescence markers (21). Experiments on mice showed that PMN also expressed VLA-4, and could inhibit the brain entry of PMN by blocking VLA-4 with antibody or down regulating VACM-1 (22). Jens Neumann et al. recorded neutrophils and microglia in the brain of mice after experimental stroke using a two-photon microscope *in vivo*. The results showed that brain resident microglia recognized both endothelial injury and neutrophil invasion. In a cooperative manner, they activate endothelial cells and capture infiltrated neutrophils. Interestingly, systemic blockade of very late antigen-4 immediately and effectively inhibited neutrophil endothelial interaction and brain entry of neutrophils (22). These results further explain the importance of neutrophils in the invasion of brain regions after ischemic stroke, and the importance of T cells in this process. This may be a new therapeutic target and significantly reduce ischemic tissue damage and effectively protect mice from stroke-related behavioral damage.

Importantly, an *in vivo* study revealed that CD3 and VLA-4 blockers have a significant synergistic effect in protecting mice from behavioral defects associated with experimental stroke, but such a synergistic effect is not exerted by the binding of an anti-Gr-1 or anti-Ly6G antibody to VLA-4. Based on the research results of Jens Neumann et al., VLA-4 - mediated neutrophil entry into the brain is a key influencing factor of behavioral injury after cerebral ischemia, while T cells contribute the least to the early behavioral dysfunction after stroke, but they reduce the infarct volume, indicating that only the of lesion volume may not be sufficient as a basis for nerve protection. But this does not deny the important role of T cells in the chronic recovery of ischemic stroke (22). Another study showed that in terms of mechanism, Treg-derived osteopontin played a role through integrin receptors on microglia to enhance the repair activity of microglia, thereby promoting the formation of oligodendrocytes and the repair of proteins. After stroke, the number of Treg cells was increased by the delivery of IL-2: IL-2 antibody complex, which improved the integrity of white matter and saved nerve function for a long time. Other studies have found that regulatory T cells can directly promote myelin regeneration (23). These results indicate that T cells have little effect on acute injury after cerebral ischemia, but play an important role in chronic recovery. Furthermore, VLA-4-mediated neutrophil entry into the brain has been confirmed by other studies (24), and neutrophils invading the brain have been shown to form extracellular DNA traps, which directly damage neurons in the CNS (25), or to induce thrombosis (26) (**Figures 1** and **4**). The importance of neutrophils in stroke outcomes has recently been demonstrated in transgenic mouse models. The study also suggested that neutrophil expression of CD39 (a cell surface ATPase) inhibits neutrophil invasion into the brain parenchyma, leading to a significant reduction in behavioral deficits after stroke (27).

Mechanistically, leukocytes may aggravate ischemic injury in many ways. First, white blood cells adhere to endothelial cells and prevent red blood cells from flowing through microvessels, which leads to the aggravation of cerebral ischemia (28, 29). Second, activated leukocytes produce proteases, MMPs and ROS, which can significantly damage the vascular endothelium and increase the permeability of the BBB (**Figure 1**). In addition, activated leukocytes can produce bioactive substances, such as leukotrienes, prostaglandins and platelet-activating factors, which can cause vasoconstriction and platelet aggregation. Finally, infiltrating leukocytes aggravate neuronal damage by activating proinflammatory factors in and around the infarct core (30). Through a series of motor coordination tests, anti-Ly6G antibody-mediated neutrophil depletion and an anti-VLA-4 antibody were shown to block neutrophil entry into the brain, thus continuously alleviating motor coordination deficits in mice subjected to transient MCAO. In contrast, in the same study, T cell depletion was shown to not cause this change in motor coordination, although it did reduce the infarct volume (31). In conclusion, these findings suggest that neutrophils have a significant adverse effect on brain remodeling and plasticity, thus exacerbating brain injury. 

## Different Neutrophil Phenotypes and Their Spatiotemporal Characteristics After Ischemic Stroke

Various animal and clinical studies have shown that in addition to activating microglia in the ischemic brain, the infiltration of circulating cells (such as granulocytes, neutrophils, monocytes/macrophages and T cells) can aggravate cell death after ischemia. In the acute phase, damaged tissues release ROS and proinflammatory factors, such as chemokines and cytokines, which induce the expression of adhesion molecules on leukocytes and brain endothelial cells and then promote the adhesion and trans endothelial metastasis of leukocytes (32). After the acute phase and in the subacute phase, the infiltrated leukocytes further release cytokines, chemokines and, more importantly, excessive ROS, which promote the production of MMPs, especially MMP-9. In addition, astrocytes usually secrete MMP-2 from the end, which acts on adjacent structures. MMP-9 and MMP-3 are produced by ECs, especially microglia and pericytes, which are the main sources of MMP-3. Neutrophils are the source of MMP-8. Therefore, MMP production is not limited to neutrophils (33). Studies by different groups have shown that MMPs activate the inflammatory response, leading to BBB destruction, brain edema, neuronal death, and hemorrhagic transformation due to overactivation of proteases and resident immune cells, and further enhance leukocyte infiltration (34) (**Figure 2**). However, MMP-9 not only plays a proinflammatory role in early ischemic brain injury but also plays an important role in brain regeneration and neurovascular remodeling, which reflects the complexity of the interaction between proinflammatory factors and tissues (35).

Similar to microglia and blood-derived macrophages, neutrophils are some of the most important white blood cells (36). The number of neutrophils in the ischemic brain reaches a peak on days 1-3 and then gradually decreases with time (2). At 48 and 96 hours after cerebral ischemia, the percentage of neutrophils found in the brain parenchyma was higher than at 14 hours. And their findings suggest that most ischemic mice have neutrophils around the pia mater and blood vessels, but only some have neutrophils in the brain parenchyma. After ischemia-reperfusion, neutrophils reach different brain septa after leaving the blood circulation, and may release intracellular components during infiltration. Then, neutrophils were detected in the perivascular space between endothelial cells and basement membrane (37). In summary, a large number of neutrophils were seen around the pia mater and blood vessels on the 4th day after ischemia. In some severely injured mice, neutrophils reached the ischemic brain parenchyma, and showed time-dependent changes in the distribution from within and around the blood vessels to the parenchyma. This effect is related to the loss of basement membrane integrity (38).

Similar to M2 macrophages, N2 neutrophils are thought to promote a reduction in inflammation by releasing anti-inflammatory mediators (39). A previous study explored the role of the peroxisome proliferator-activated receptor gamma (PPAR-γ) agonist rosiglitazone in C57BL6 mice subjected to permanent peripheral MCAO. In this study, neutrophils were found to adopt the N2 phenotype and express M2 markers, namely, Ym1 and CD206. Importantly, rosiglitazone was shown to increase the phagocytosis of neutrophils by microglia/macrophages, preferentially affecting N2 neutrophils. The switch of neutrophils to the N2 phenotype during differentiation is related to alleviation of cerebral infarction, which is prevented by neutrophil depletion (40). These results suggest that N2 neutrophils have neuroprotective effects in the ischemic brain. Specifically, pro-inflammatory neutrophils secrete chemokines such as CCL2 (monocyte chemoattractant protein-1) and CCL17, which recruit monocytes and T regulatory cells, respectively (41). The secretion of IL-8 causes more neutrophils to be recruited to inflammatory sites. In addition, Oncostatin M is a member of the IL-6 family, secreted by neutrophils, which has a variety of pro-inflammatory and angiogenesis effects, including inducing neutrophil adhesion and chemotaxis, increasing endothelial cells to produce chemokines, stimulating vascular endothelial growth factor production (42).

Studies have shown that Toll-like receptor 4 (TLR4) deficiency increases the number of neutrophils (N2), which is related to neuroprotection after stroke, suggesting that the regulation of neutrophil polarization is the main target of TLR4 and emphasizing the key role of TLR4 in the prognosis of stroke (43). It has also been found that the loss of TLR4 in neutrophils regulates the induction of several pathways previously associated with inflammatory processes after ischemia, all of which may be the basis of neurological outcome during stroke (44). TLR4 deficiency not only affects the differentiation of neutrophils, but also affects the activation of microglia and macrophages (45). Another study showed that G protein-coupled receptor 30 (GPR30) was highly expressed in microglia and increased significantly after ischemic injury. The activation of GPR30 reduced the activation of microglia, significantly reduced infarct volume, improved neurological deficits and reduced neuronal damage. The mechanism is that GPR30 activation reduces the expression of Iba1 and TLR4 protein and TLR4 mRNA level, and inhibits the activity of NF-κB (46).

Generally, neutrophils are mainly harmful, and they affect prognosis, severity and the infarct volume by exerting their effects, including inducing the no-reflow phenomenon; releasing elastase, which may increase tissue damage; and producing reactive oxygen species (ROS), which can damage the blood-brain barrier (BBB) (47) **(** **Figure 2** **)**. Consistent with the harmful effects of neutrophil infiltration, neutrophil depletion in WT animals results in a significant reduction in infarct size, which is consistent with previous data (48). However, when combined with TLR4 deletion, depletion of neutrophils not only does not induce neuroprotection but also exacerbates brain injury after stroke. Thus, neutrophils do not play a harmful role in the absence of TLR4, and the neuroprotective effect of TLR4 deletion requires neutrophils (49). Neutrophils may respond to different molecules, such as cytokines (TGF-β, IL-27), injury-related molecular patterns and growth factors released after stroke, which have been reported to be related to neutrophil polarization (50). At present, it is believed that some neutrophil subsets show different characteristics, with the characteristics of anti-inflammatory, angiogenesis or dissolution. For example, the presence of IL-10, TGFb, IL-13 and IL-4 will induce N2 polarization, while the presence of IFNb, IFNg and IL-12 will facilitate N1 polarization (51). TGF β Induced differentiation of neutrophils into N2 phenotype (52). TGF β is mainly up-regulated in microglia and macrophages after ischemia a profound impact on immune cells in addition to its neuroprotective properties. Despite known pro-inflammatory effects, TGF-β inhibits inflammation by inhibiting Th1 and Th2 responses and promoting Treg cell development (41). Therefore, the production of TGF-β、IL-10 after cerebral ischemia promotes tissue repair by promoting the regression of inflammation and the direct cell protection of surviving cells in ischemic area (53). In conclusion, TLR4 plays an important role in the phenotypic polarization of neutrophils after stroke, suggesting that TLR4 deletion is related to an increase in the number of N2 neutrophils in such a proinflammatory environment, which may be related to the neuroprotective effect observed in these mice.

## Evolution and Migration of Neutrophils From the Periphery, Where They Are Produced, to the Brain

### Production of Neutrophils

The production of neutrophils mainly depends on the hematopoietic activity of bone marrow. Bone marrow is responsible for two-thirds of all hematopoietic activity in adults. Hematopoietic stem cells are located in the niches formed by osteoblasts, which are characterized by low blood flow and low oxygen tension, while mature and active proliferating stem cells are located on the side near the edge of sinusoids, which are special vascular structures of bone marrow (54). In a study on peripheral bone marrow hematopoietic stem cells after ischemic stroke, increased sympathetic nervous system signals activate hematopoietic stem cell activity in bone marrow and increase the output of neutrophils and inflammatory Ly-6Chigh monocytes after stroke (55). Adam Denes et al. evaluated whether experimental stroke caused the activation of leukocytes in bone marrow by evaluating two common signaling pathways NFκB and p38MAPK in different hematopoietic cells. They identified the rapid induction of NFκB p65 in bone marrow after 70 min of ischemia by western blotting and further enhanced it for 4 h. Single cell analysis by flow cytometry showed that NFκB p65 phosphorylation occurred in myeloid cells after stroke, mainly in Gr-1 positive granulocytes. Compared with the sham operation group, the phosphorylation of NFκB p65 in granulocytes was significantly increased after 4 h reperfusion after stroke. Bone marrow is the main source of circulating monocytes and granulocytes (56). The release of CXCR2-positive granulocytes in bone marrow at the early stage of cerebral ischemia (4 hours) is related to the rapid systemic up-regulation of CXCL1 (CXCR2 ligand) and granulocyte colony-stimulating factor (key cytokines involved in bone marrow leukocyte mobilization). This process involves the rapid activation of NFκB and p38 mitogen-activated protein kinases in marrow-like cells (57). David Weisenburger-Lile et al. reported that the inflammatory characteristics of circulating neutrophils increased during ischemic stroke (IS), which was related to the expansion of harmful neutrophil subsets. The percentage of neutrophils with reverse transendothelial migration (CD54^high^CXCR1^low^) phenotype increased. These changes in neutrophil homeostasis are associated with the severity of the disease and may play an important role by causing systemic inflammation and breakdown of the blood-brain barrier (58). And another study shows that, in the first week after ischemic stroke, the number of monocytes expressing IL-8 mRNA in circulation increased significantly, and the level of IL-8 in plasma increased significantly. Because of its strong chemotaxis, IL-8 can mediate the accumulation of neutrophils in damaged tissues after cerebral ischemia (59). β-1 integrins such as a4b1, a6b1, and a9b1 are expressed on hematopoietic stem cells and interact with osteoblasts and extracellular matrix (ECM) in stem cell niches (60, 61). The chemokine receptor CXC motif receptor (CXCR)4 is essential for stem cells and more mature neutrophils to home to bone marrow (62). CXCR4 binds to CXCL12, which is expressed by bone marrow stromal cells, including osteoblasts and vascular endothelial cells in bone marrow (63, 64).

The production of neutrophils is widespread under stable conditions. Normal adults produce 1~2 × 10^11 cells per day. Granulocyte colony-stimulating factor (G-CSF) is needed to regulate the production of neutrophils to meet the need for an increased number of neutrophils during infection, but G-CSF is not absolutely necessary for granulopoiesis; in G-CSF knockout mice, approximately 25% of the normal number of residual granulocytes is generated, and mature neutrophils are produced (65). The production of neutrophils is largely regulated by the rate of neutrophil apoptosis. When macrophages and dendritic cells phagocytize apoptotic neutrophils, the production of interleukin (IL)-23 is reduced (66). IL-23 stimulates specific T cells known as neutrophil regulatory T cells, which are mainly located in mesenteric lymph nodes, to produce IL-17A, an important stimulator of G-CSF production (67). They are collectively called neutrophil regulatory T cells or Tn cells. About 60% of Tn cells were γδ T cells, about 25% were NKT-like cells, and less than 15% were CD4 T cells. IL-17A produced by Tn cells regulates G-CSF production, thereby promoting promyelocyte proliferation and neutrophil maturation (68). G-CSF is produced by mononuclear/macrophage-derived bone marrow stromal cells, vascular endothelial cells, fibroblasts and mesothelial cells, and its expression is strictly regulated. G-CSF binds to a known receptor G-CSFR, which is expressed in all neutrophil precursor cells in bone marrow, and mature neutrophils express the most receptors on its surface. When G-CSF binds to its receptor, it causes the proliferation, differentiation and activation of granulocyte precursors (68). A physiological feedback circuit, IL-23 – IL-17 – G-CSF axis is the main way to regulate mouse neutrophil homeostasis (69). Therefore, the production of G-CSF decreased with the increase of apoptotic neutrophils in tissues.

### The Inflammatory Complex Stimulates the Differentiation of Premature Bone Marrow-Derived Granulocytes

The inflammatory complex is a potential major regulator of hematopoiesis, which involves a combination of cholesterol and glucose metabolism and myeloid differentiation. During the process of granulopoiesis in the context of chronic inflammation, inflammatory cells play a central role in myeloid differentiation because caspase 1-dependent cleavage of the transcription factor GATA1 promotes the production of neutrophils, while red blood cell differentiation is impaired (70). Therefore, patients with chronic inflammatory diseases often have anemia. In addition, IL-1β directly stimulates the differentiation of premature granulocytes by activating a PU.1-dependent gene program. Regulation of IL-1β signal-dependent hematopoietic stem and progenitor cell (HSPC) proliferation is achieved in these cells through regulation of glucose and cholesterol metabolism, which is a key process in immune training (71). In contrast, hypercholesterolemia mediates the long-term proliferation of bone marrow progenitor cells and enhancement of the immune response in these cells in an NLRP3 inflammasome-dependent manner (72). In addition, inhibition of cholesterol efflux from neutrophils can also increase the activation of NLRP3 inflammatory bodies, promote neutrophil infiltration and release NETs, thus accelerating the formation of atherosclerotic lesions. Therefore, these results provide evidence for the role of inflammatory corpuscles and cholesterol metabolism in the regulation of neutrophil activation, not just bone marrow hematopoiesis (73).

### Neutrophils Are Released From Bone Marrow

An increase in the number of neutrophils is positively correlated with infarct size and functional defects (74). CXCR4 plays an important role in maintaining bone marrow neutrophils. The absence of CXCR4 can lead to the migration of mature neutrophils from bone marrow to the circulation without affecting the life span of circulating neutrophils (63). In contrast, mutations in CXCR4 lead to increased signaling, leading to clinical syndromes characterized by a lack of neutrophils in the circulation and an increase in the accumulation of mature neutrophils in the bone marrow (verruca, hypogammaglobulinemia, infection, and myelodysplasia) (75). CXCR2 is another cytokine receptor expressed on myeloid cells. CXCL1 and CXCL2 (KC and Grob or MIP-2, respectively) are expressed by bone marrow endothelial cells. The deletion of CXCR2 can also lead to a myeloproliferative phenotype, with mature neutrophils remaining in the bone marrow, while deletion of both CXCR2 and CXCR4 results in a similar phenotype as CXCR4 deletion; that is, neutrophils cannot be retained in bone marrow (57). Therefore, CXCR4 is necessary for retaining neutrophils in bone marrow. Release of neutrophils may be affected by CXCR2, G-CSF receptor (G-CSFR), or TLR signaling, all of which occur in the late stages of bone marrow neutrophil maturation (76). CXCR4 signaling mainly regulates the transport of bone marrow neutrophils, and disruption of CXCR4 signaling may be a common mechanism of cytokine- and chemokine-induced release of bone marrow neutrophils (77). The expression of SDF-1 is downregulated by G-CSF, while the expression of KC and Grob is upregulated by G-CSF, making CXCR4-CXCR2 signal transduction beneficial for the release of CXCR2 and neutrophils (57). These data suggest that drugs that regulate CXCR4 signaling may be effective in controlling neutrophil responses in infectious and inflammatory diseases.

### Chemotactic Movement of Neutrophils Into the CNS

In the ischemic core area, a sharp decrease in cerebral blood flow leads to the rapid death of neurons followed by the release of DAMPs, which promote the activation and proliferation of local microglia (resident macrophages of the CNS) (78). Although they are vulnerable to the adverse effects of ischemic injury, microglia can be polarized toward the M2 phenotype, which is an anti-inflammatory phenotype characterized by phagocytic activity, which contributes to debris clearance and tissue repair (79, 80). In addition, activated microglia release proinflammatory mediators, such as cytokines, TNF and ROS, causing BBB damage (81), thus promoting the entry of leukocytes (including monocytes, macrophages, neutrophils and lymphocytes) into the brain (82). Inflammatory signals in macrophages are very powerful and complex. Once macrophages infiltrate the ischemic brain, they express high levels of proinflammatory cytokines, such as IL-1β (32).

Acute ischemic stroke is a hypoxic-ischemic disease related to aseptic inflammatory response (83). Ischemic injury triggers immune responses that promote the migration and infiltration of immune cells into the brain parenchyma in a coordinated time pattern (84). Due to the significant destructive potential of these cells, neutrophils have received special attention for many years, either through the direct neurotoxicity caused by the release of proteolytic enzymes (85), or through the indirect effect of neutrophil aggregation in blood vessels, blocked capillary blood flow and no reflow phenomenon (86). Isabel Perez−de−Puig et al. used immunofluorescence and flow cytometry to study the recruitment process of brain neutrophils in two mouse models of permanent ischemia induced by distal ligation of middle cerebral artery (c- MCAo) or intraluminal MCA occlusion (il- MCAo) (30). The results showed that during MCAo, neutrophils exuded from the perileptomeningeal blood vessels around the infarcted tissue and reached the cortical parenchymal basement membrane and the perivascular space of the cortical arterioles, reaching the cortical area without blood flow. After long-term ischemia, neutrophils are activated, showing signs of forming a network structure in the lumen and around the blood vessels, indicating that neurovascular units are the targets of neutrophils in stroke. In addition, they investigated the brains of three stroke patients, all of whom died within 1 – 5 days after stroke. The anatomical localization of neutrophil infiltration in fatal human stroke patients was evaluated in the brain tissue after death, which confirmed that neutrophils were indeed recruited to the central part after ischemic stroke and participated in the subsequent pathophysiological process (30).

### Effect of Neutrophils on BBB Damage and Intracerebral Hemorrhage

The relationship between neutrophil count and the NLR in the peripheral blood of patients with ischemic stroke, cerebral hemorrhage and cerebral edema indicates that the effect of neutrophils on the microvasculature leads to the destruction of BBB (87). In an immunohistochemical study of patients with fatal ischemic stroke, MMP-9-positive neutrophils were shown to infiltrate around the cerebral microvessels, resulting in the loss of type IV collagen in the basement membrane and the formation of cerebral hemorrhage, which indicates that the degradation of ECM mediated by MMP-9 may be related to hemorrhagic transformation (88) (**Figures 1** and **2**). In Sprague-Dawley rats with type IV collagenase-induced intracerebral hemorrhage, neutrophils were shown to induce BBB disruption (34). In this study, neutrophil depletion induced by anti-neutrophil antibodies reduced BBB leakage in the perihematomal area. The expression of MMP-9 decreased significantly due to neutrophil depletion. The tight junction proteins (TJPs) combine with ECs to form a blood-brain barrier. In the brains of rodents and adults, ZO-1, claudin-1, claudin-5 and occludin were found to exist in the tight junction of the brain endothelium forming BBB (89). Occludin, claudin-5 and ZO-1 are the main structural barrier proteins of BBB, which are considered to be sensitive indicators of normal and disordered functional status of BBB. In the reperfusion model, the increase of MMP-2 at the early stage of injury was observed in rodents and non-human primates (90). Treatment with MMP inhibitor or neutralizing antibody can reduce infarct size and prevent BBB damage after focal ischemic stroke (91, 92). These findings have also been supported by previous studies showing that chronic BBB breakdown is associated with a decrease in the number of microvessels (93). In conclusion, neutrophils play an important role in the destruction of the BBB in ischemic stroke due to the destruction of microvessels.

## The Possible Mechanisms by Which Neutrophils Play Different Roles After Ischemic Stroke

### Neutrophil-Mediated Excessive Production of ROS Aggravates Neurological Damage After Ischemic Stroke

In healthy individuals, circulating neutrophils are dormant, and the efficiency with which endothelial cells capture and block them is very low. Subsequently, inflammatory mediators and neutrophils are released across the endothelial barrier during infection (94). Phagocytosis triggers the activation of neutrophils, resulting in the release of antimicrobial peptides, proteases, MPO and $O_{2}^{-}$ from phagocytes (95). $O_{2}^{-}$ is produced by the activation of NADPH oxidase. $O_{2}^{-}$ is the “blasting fuse” of ROS, namely, hydrogen peroxide (H_2_O_2_), hydroxyl radical (OH-) and hypochlorite (HOCl) (96) (**Figure 2A**). ROS produced by the phagocyte NADPH oxidase NOX2 play a key role in host defense against microbial pathogens. However, excessive ROS release can also damage surrounding host tissues, thereby amplifying the inflammatory response (**Figure 2B**).

The accumulation of neutrophils and the release of ROS from inflammatory sites are believed to contribute to tissue damage in inflammatory diseases. At inflammatory sites, high levels of proinflammatory cytokines such as GM-CSF and TNF α can enhance the release of ROS, and anti-TNF α therapy is very beneficial in RA (97), As (98), Crohn’s disease (99) and other autoimmune diseases and infectious diseases. In vitro data have shown that ROS increase endothelial-neutrophil interactions (100), TNF-induced fMLF stimulates neutrophil activation to increase microvascular permeability (101), and the activation of neutrophils is related to neutrophil-mediated endothelial cell injury (102). In animal models, TNF induces neutrophils to interact with intravascular immune complexes and to adhere to endothelial cells (103). Neutrophil-derived ROS mediate endothelial cell injury and organ inflammation (104). In addition, neutrophil respiratory burst initiation predicts capillary leakage (105) and mediates inflammation in rats (7). In an *in vitro* model, cells from severe sepsis patients showed neutrophil promoter groups, and the initiation of neutrophil respiratory burst by TNF or GM-CSF has been observed in human subjects. These data suggest that neutrophil-derived ROS can induce tissue damage *in vitro* and *in vivo* by activating NADPH oxidase (**Figure 2B**). Therefore, there is convincing evidence that neutrophil respiratory burst, which also aggravates neurological damage after ischemic stroke, is associated with human inflammatory diseases (106).

### Neutrophils Accelerate Atherosclerosis

**Figure 3:**
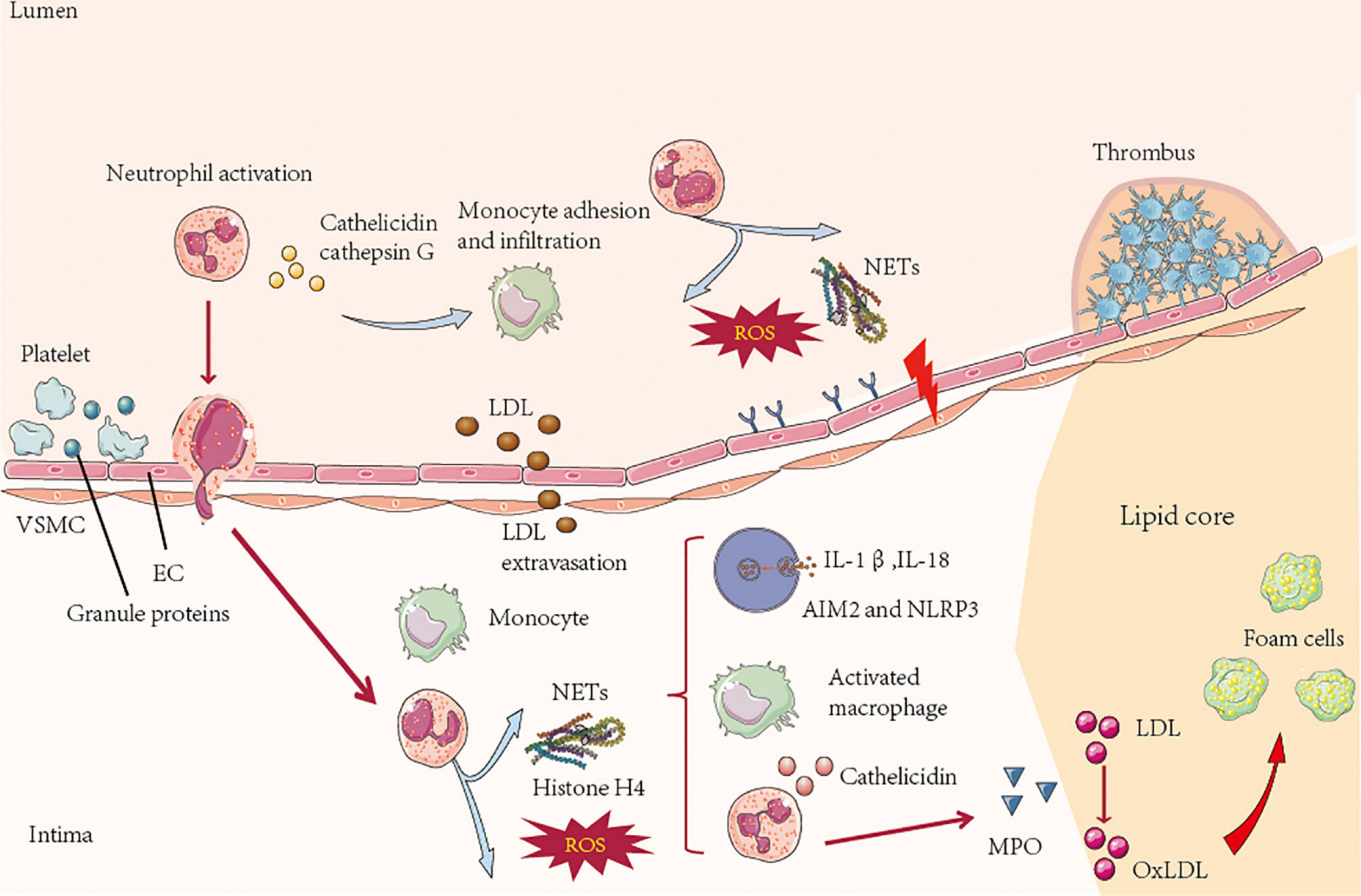
In the process of atherosclerosis, platelet-derived chemokines, such as CC chemokine ligand 5, promote the activation and recruitment of neutrophils. On the lumen side, activated neutrophils secrete granule proteins, including cathepsin G, which directly or indirectly promote the recruitment of myeloid cells. ROS and proteases secreted by neutrophils in the lumen and intima of atherosclerotic plaques lead to activation and dysfunction of the extracellular matrix in the layer and bottom of endothelial cells (ECs), resulting in leukocyte infiltration and low-density lipoprotein (LDL) extravasation. In the process of atherosclerotic plaque formation, the neutrophil-derived granule proteins antimicrobial peptides and α-defensin activate macrophages and cause them adopt a proinflammatory state. Neutrophils secrete MPO, which mediates the oxidation of low-density lipoprotein (oxLDL) and promotes the formation of foam cells. NETs stimulate plasma-like dendritic cells (pDCs) to produce atherogenic interferon (IFN)-α through NLRP3, and macrophages produce IL-1β and IL-18 but not AIM2 In the late stage of atherosclerosis, neutrophils can destroy plaque stability by secreting a network of proteins including cytotoxic histone H4, which penetrates and eventually dissolves vascular smooth muscle cells (VSMCs). Neutrophil-derived metalloproteinases can also induce VSMC death by degrading ECM. The death of VSMCs and degradation of ECM lead to the thinning of fiber caps and the formation of vulnerable plaques. Neutrophils can trigger epithelial cell desquamation, which is specifically manifested by the stimulation of endothelial cell stress and apoptosis by neutrophils, leading to endothelial cell detachment. This process is regulated by Toll-like receptor 2 (TLR2) signaling and other neural network signals in endothelial cells.

Atherosclerosis is the pathological basis of myocardial infarction and stroke, which have become major causes of death worldwide (107). A prospective study showed that there was a significant interaction between carotid atherosclerotic plaque and VIM (vimentin) in the incidence of ischemic stroke. After stratified by carotid plaque, high VIM had a stronger correlation with stroke in carotid plaque participants, especially the risk of ischemic stroke (108). Sepideh Amin-Hanjan et al. studied the hemodynamic stability over time and its association with stroke risk in a prospective cohort study of VERiTAS (Vertebrobasilar Flow Evaluation and Risk of Transient Ischemic Attack and Stroke). The results showed that the risk of stroke in patients with recently symptomatic vertebrobasilar atherosclerosis disease and posterior circulation hemodynamic injury after one year was five times that in patients without hemodynamic injury (22% vs 4%) (109). Therefore, there is an important correlation between atherosclerosis and ischemic stroke, and neutrophils play an important role in this pathophysiological process. Atherosclerosis is characterized by arterial wall injury driven by an unbalanced lipid profile, oscillatory shear stress and proinflammatory cytokines. Activation of arterial endothelial cells leads to the adhesion of myeloid cells to endothelial cells and their infiltration of the arterial intima. The accumulation of immune cells, lipoproteins and cell debris in the arterial intima leads to the aggregation and instability of atherosclerotic plaques (110). Neutrophils promote monocyte recruitment (111). Cathepsin G, cathepsin- or neutrophil-derived α-defensin and the platelet-associated CCL5 complex are immobilized on endothelial cells and can induce monocyte adhesion in a mouse vascular inflammation model (112). In the initial stage of atherosclerosis, the imbalance between and activation of the endothelial layer and the disintegration of the underlying ECM increase the adhesion and transfer of immune cells and induces the transfers LDL from the lumen side to the basal surface. Neutrophil granule proteins, such as neutrophil-derived azurol, proteinase-3 and α-defensin, can increase the surface expression of endothelial cell adhesion molecules and regulate the permeability of endothelial cells (113). In addition to regulating the entry of monocytes and macrophages, they also regulate other factors that promote atherosclerosis (**Figure 3**). For example, the production of hydrochloric acid by MPO can lead to LDL oxidation, thereby accelerating the formation of foam cells (114). Furthermore, the protein granulosa directly activates human and mouse macrophages, induces proinflammatory signals in macrophages and promotes the secretion of cytokines, which have been proven to promote atherosclerosis. NETs can drive atherosclerosis by activating macrophages. In atherosclerotic mice, NETs can stimulate macrophages to produce IL-1β by stimulating NLRP3 inflammatory bodies (16) (**Figure 3**).

Neutrophils promote endothelial desquamation of atherosclerotic plaques. In human specimens, neutrophils colocalize with TLR2-expressing plaques on endothelial cells. The stimulation of TLR2 on endothelial cells leads to endothelial cell stress and apoptosis, which is enhanced in the presence of neutrophils (115). Similar results have been found in a newly established mouse model of endothelial cell erosion (101). In this model, neutrophil adhesion to endothelial cells is negatively correlated with endothelial continuity, which is regulated by TLR2-dependent endothelial cell activation. These effects are abolished by TLR2 deficiency, neutropenia, or neutrophil adhesion arrest, suggesting that neutrophils are involved in endothelial cell invasion (116) (**Figure 3**). In conclusion, neutrophils are involved in the induction and development of atherosclerosis.

### The Role of NETs in Ischemic Stroke

**Figure 4:**
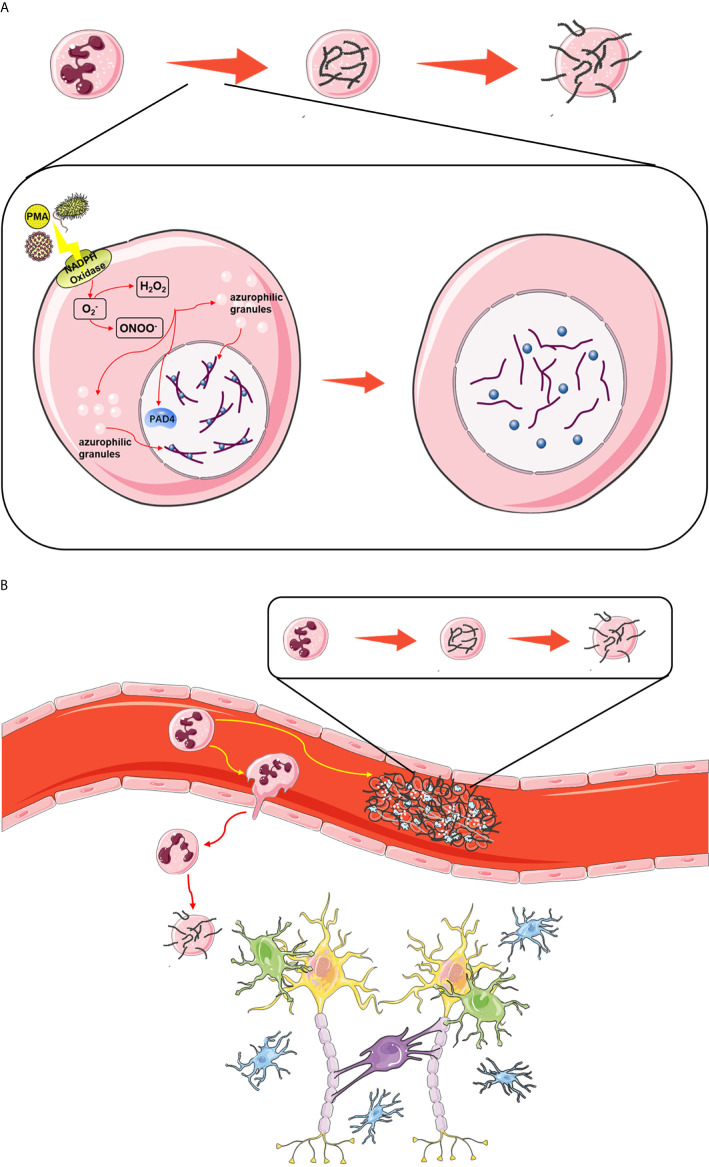
**(A)** The classic pathway of NETosis. Upon stimulation, ROS produced by NADPH oxidase activate the binding of PAD4 to citrullinated histones, leading to the unfolding of chromatin, and induce the translocation of enzymes such as NE and MPO in azurophilic granules to the nucleus, where they exacerbate the dissociation of histones and DNA. Finally, after the nuclear membrane and cytomembrane are dissolved, cell components including DNA, histones and granzymes are released into the intercellular space. **(B)** The effect of NETosis in ischemic stroke. Upon stimulation, neutrophils in the thrombus release NETs, which act as scaffolds to consolidate the thrombus. Some circulating neutrophils migrate from the blood stream into brain tissue, and they also release NETs to exacerbate neural damage.

NETs are large, extracellular, reticular structures composed of cytoplasmic proteins and granular proteins assembled on a de-aggregated chromatin scaffold. Although most of the DNA in NETs originate from the nucleus, these structures also contain mitochondrial DNA. NETs capture, neutralize and kill bacteria, fungi, viruses and parasites and are considered to prevent bacterial and fungal transmission. However, if the regulation is disordered, NETs can contribute to the pathogenesis of immune-related diseases (117). NETs are produced as follows. High mobility group box 1 (HMGB1) is a danger associated molecular pattern (DAMP) molecule (118). Seung woo Kim et al. showed that a large amount of accumulation in serum after permanent MCAO played a key role in CitH3 induction of brain parenchymal neutrophils and peripheral blood neutrophils. The all-thiol and disulfide types of HMGB1 induce CitH3 through its specific receptors CXCR4 and TLR4 respectively. Importantly, HMGB1 not only induces NETosis, but also is included as a part of squeezing NETs, which contributes to NETosis mediated neuronal death. Therefore, there is a vicious cycle between neuronal cell death and NETosis, and HMGB1 mediates the harmful effect of this cycle (119). After MCAO animals were treated with PAD inhibitor to inhibit NETosis, delayed immune cell infiltration was significantly inhibited, and vascular injury was significantly reduced (120). NETosis, mediated by HMGB1, aggravates inflammation and subsequent damage in ischemic brain tissue (5). After neutrophils are stimulated, they undergo the following changes at the cellular level: elimination of lobulated nuclei, chromatin deconcentration, separation of the inner and outer nuclear membranes, disintegration of cytoplasmic granules, and destruction of nuclear membranes. Finally, chromatin is excreted with cytoplasmic granules from the cell (121). At the molecular level, phorbol 12 myristate 13 acetate (PMA; a protein kinase C activator extracted from plants) can activate NADPH oxidase and then induce ROS production in cells. ROS can induce the activation of protein arginine deiminase 4 (PAD4). Under the action of PAD4, the arginine residues of histones on chromatin become citrullinated, causing them to lose the ability to firmly bind to the DNA strand (122). Then, the hinge is opened and becomes loose, chromatin disintegrates, histones fall off, and the DNA becomes naked (**Figure 4A**). At the same time, elastase and MPO in characteristic cytoplasmic azurophilic granules of central granulocytes enter the nucleus through the broken nuclear membrane, further destroying the integrity of the chromosome and causing it to disintegrate (5). Finally, DNA strands, histones, granule proteins and cytoplasmic proteins are secreted together into the ECM, resulting in the formation of a three-dimensional network structure based on the DNA chain network and attached by a variety of intracellular effluents (123) (**Figure 4B**).

In addition, NETs promote the growth of thrombi, thus increasing the stroke area. Finally, neutrophils increase neural cell death, a process likely involving NETs. Mechanistically, neutrophils can amplify thrombosis through the cleavage of coagulation factors and the activation of platelets (124) (**Figures 3** and **4B**). Cathepsin G promotes firm adhesion and exosmosis of neutrophils and monocytes in arterial endothelial cells (125). The number of neutrophils that produce NETs in blood vessels and the brain parenchyma reaches a peak at 3-5 days. Neutrophil depletion can reduce damage to the BBB and promote neovascularization 14 days after stroke. PAD4 is an enzyme essential for the formation of reticular structures, and its expression is upregulated in the brain after ischemia. Overexpression of PAD4 promotes the formation of reticular structures, decreases neovascularization and increases BBB injury (126). In addition to bacterial death, activation of neutrophils also results in the release of nuclear and granular contents, which form a wide network of DNA complexes (NETs, reticular networks) (127). Reticular structures contain double-stranded DNA, histones and granins, including neutrophil elastase (NE), cathepsin G and MPO (123). These reticular networks are associated with autoimmune diseases (128), cardiovascular and pulmonary diseases (129), and inflammation (130), which are related to thrombosis (131).

Studies have shown that neutrophils isolated from ischemic mice form more spontaneous reticular structures and show a greater tendency to form reticular structures after lipopolysaccharide (LPS) exposure. In summary, the data suggest that stroke-activated neutrophils release excessive reticular networks into blood vessels and the parenchyma, which is consistent with elevated levels of circulating DNA (5). In a previous study, circulating DNA levels were found to be elevated and to peak 3-5 days after cerebral ischemia. Neutrophil-dependent net formation was observed in blood vessels and brain parenchyma around the infarct. Digestion of NETs with DNase#I#can significantly reduce BBB injury and increase microvascular cell coverage and the formation of new functional vessels; this finding suggests that the formation of NETs is the cause of vascular injury (5). It has also been proven that neutrophils and reticular structures can improve cerebral vascular remodeling and functional recovery in the delayed stage after stroke (5).

Stroke leads to upregulation of STING expression, activation of TBK1 and IRF3, and induction of IRF3-dependent IFN-β synthesis. Previous studies have shown that free DNA can combine with cyclic GMP-AMP synthetase to promote the synthesis of STING-dependent IFN type I (132, 133). STING (STimulator of INterferon Genes) is considered to be necessary to control the host defense strategy caused by cytoplasmic DNA and CDNs. Chronic STING activation may also be the cause of some inflammatory diseases manifested by its own DNA. Studies have shown that circulating GMP-AMP synthase (cGAS) plays a key role in STING activation. Silencing of STING and an IFNAR antibody can promote angiogenesis and repair in mice. In addition, it was found that the PAD inhibitor CL amidine inhibits the activation of the STING pathway and the production of IFN-β. Therefore, the type I IFN response may be related to reticular structures and ischemic vascular remodeling (5, 134). In conclusion, it is important to increase the clearance of NETs, which regulate harmful vascular remodeling and promote vascular repair after stroke. Reticular structures are considered key targets for promoting stroke-mediated neovascularization and functional recovery.

### Neutrophils Regulate Endothelial Cell Function in the Repair Process After Ischemic Stroke

**Figure 5:**
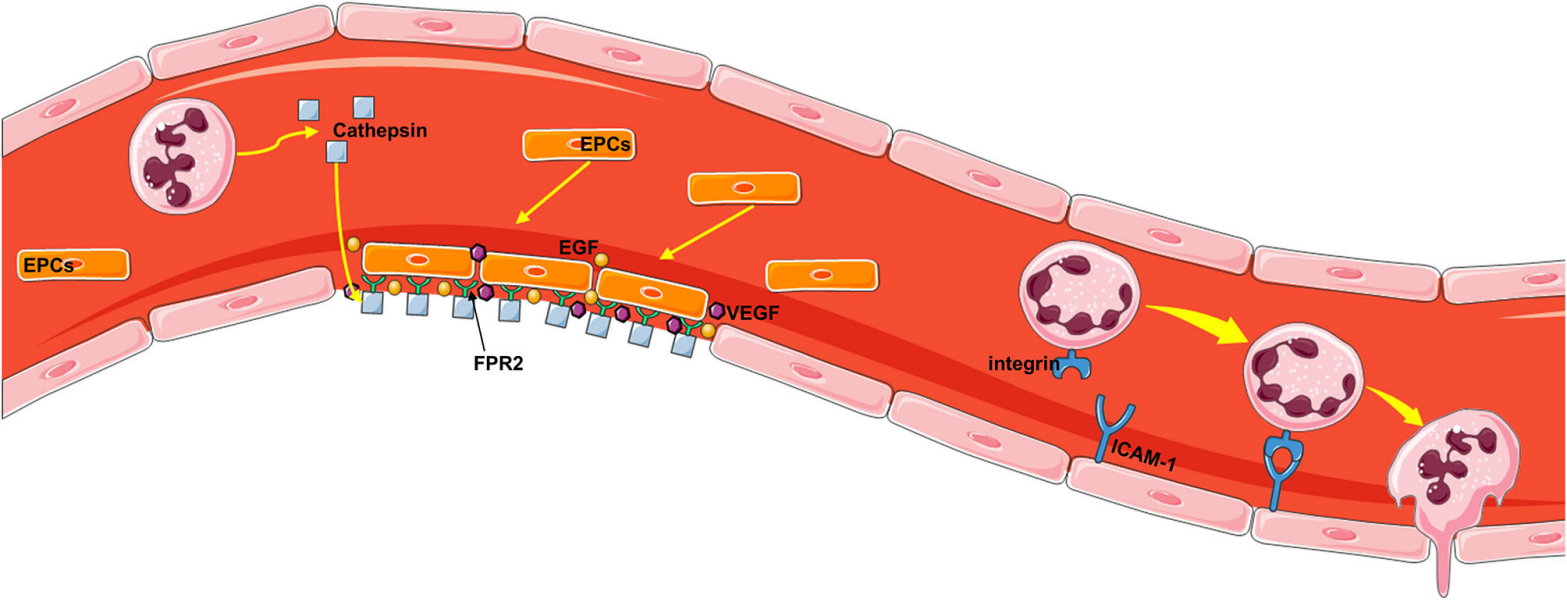
The mechanism by which neutrophils repair the vascular endothelium. First, activated neutrophils deposit the antimicrobial peptide cathelicidin, which promotes the adhesion of circulating endothelial progenitor cells through FPR2, at the site of arterial injury. Endothelial progenitor cells recruited in this way can directly cover the injury site but also release angiogenic growth factor in a paracrine manner, thus promoting reendothelialization. Cathelicidin stimulates endothelial progenitor cells to secrete VEGF and EGF, which can promote the repair of damaged endothelial cells. Neutrophils penetrate the vascular endothelium by reacting with endothelial adhesion molecules to reduce blood flow velocity in the vasculature, and then neutrophils cross the vascular wall with the help of a series of adhesion molecules, such as P-, E-, and L-selectin; ICAM-1; and integrins (CD11b, a, and c).

The core pathophysiological mechanism of neutrophil-driven repair is angiogenesis, which is an important process by which nutrients and oxygen are delivered to healing tissue. The mechanism of how neutrophils are directly involved in endothelial repair has been partially explained in studies of neutropenic mice and mice lacking cationic antimicrobial peptides with chemotactic properties. Cathepsin deposition along the injured arterial lumen induces activation of circulating endothelial progenitor cells in an FPR2-dependent manner (135). Endothelial progenitor cells recruited in this way promote re endothelialization by directly covering the injured site but also releasing angiogenic growth factor in a paracrine manner (136) (**Figure 5**). At the site of arterial injury, activated neutrophils deposit cathelicidin antimicrobial peptide, which promotes the adhesion of circulating endothelial progenitor cells through FPR2. Cathelicidin stimulates endothelial cell progenitors to release VEGF and EGF in a paracrine manner. These two processes complement each other to promote the recovery of endothelial cells (137). In addition, previous studies have shown that neutrophils penetrate the vascular endothelium by reacting with endothelial adhesion molecules to reduce blood flow velocity in blood vessels, and then neutrophils cross the BBB with the help of a series of adhesion molecules, such as P-, E-, and L-selectin; ICAM-1; and integrins (CD11b, a, and c) (**Figure 5**).

We recognize that according to some points of view, the role of neutrophils in ischemic stroke is two-sided. The proinflammatory characteristics of neutrophils may further aggravate cerebral ischemic injury. A study showed that infiltrating neutrophils exert harmful effects by releasing oxygen free radicals, proteases and proinflammatory cytokines, which can aggravate inflammatory damage (2). Neutrophil activation can activate immune cells, drive complete immune function, and promote neovascularization by repairing damaged vascular endothelium, which indicates that neutrophil infiltration also exert beneficial effects (138). MMPs have harmful effects in the early stages of ischemia, but are beneficial in the recovery phase, especially during angiogenesis and cerebral blood flow reconstruction (139). Nerve repair involves three processes: angiogenesis, neurogenesis and synaptic plasticity. After stroke, ischemic penumbra tissue releases angiogenic factors, which can form new blood vessels through endothelial progenitor cell migration and induce endothelial cell proliferation. Angiogenesis promotes neural repair, including neurogenesis and synaptic initiation (137). In addition, the factors released by ECs trigger the proliferation of neural stem cells (140). The main migration process of neural progenitor cells (NPCs) is closely related to blood vessels, which indicates that this interaction provides directional guidance for NPCs. These findings suggest that blood vessels play a key role in the migration of NPC to infarct area. Angiogenesis requires the degradation of vascular basement membrane and the remodeling of ECM in order for ECs to migrate and invade surrounding tissues (141). Long-term MMP inhibition reduces neuronal plasticity, damages new angiogenesis, and promotes cortical hemorrhage and tissue damage around infarction (142). The time distribution of MMP-9 after intracerebral hemorrhage was similar to that after stroke/reperfusion, suggesting that the increase of MMP-9 showed a bimodal pattern (88). On day 7 and 14, MMP-9 signal increased and co localized with NeuN positive and GFAP positive cells. MMP-9 expression in astrocytes was associated with ECs markers. Consistent with these findings, inhibition of MMP 7 days after stroke reduces neuronal plasticity and vascular remodeling, and increases tissue damage in the infarcted cortex (143). It is suggested that the second mode of MMP-9 elevation may involve neurovascular remodeling in the surrounding area of infarction within 7 – 14 days.

### The Role of Neutrophils in Hyperlipidemic Ischemic Stroke

Compared with WT mice, which exhibit normal blood lipid levels, hyperlipidemic ApoE-/- mice suffer from severe brain injury following focal cerebral ischemia, which is related to the increase in granulocyte counts in the spleen and blood (144, 145). Another study explored the relationship between residual cholesterol level and cIMT in patients with ischemic stroke. Common carotid intima-media thickness (cIMT) is an imaging indicator of subclinical atherosclerosis. The results showed that the elevated fasting residual cholesterol level in patients with ischemic stroke was positively correlated with the average cIMT and the maximum cIMT, even in patients with the best LDL cholesterol level (146). CXCR2 antagonist reduced neurological deficits and infarct volume in hyperlipidemic ApoE-/- mice. This effect is the same as that of neutropenia. After hyperlipidemia and ischemia, brain neutrophil infiltration and peripheral neutrophil increase, and the use of CXCR2 antagonist reduces neutrophil infiltration. The decrease of neutrophil response was related to the increase of neutrophil apoptosis and the decrease of CXCR2, iNOS and NOX2 expression in bone marrow neutrophils (147). Neutrophils produce and release (a) proinflammatory cytokines, which promote the opening of the BBB; (b) ROS, which induce structural damage to brain ECM proteins; and (c) hydrolases such as elastases, which degrade ECM proteins and proteoglycans. These effects may lead to brain injury and poor neurological recovery. The CXCR2 antagonist SB225002 regulates the expression of oxidative stress-related enzymes in bone marrow neutrophils and alleviates oxidative damage in the ischemic brains of hyperlipidemic ApoE-/- mice. Pharmacology or antibody-mediated antagonism of neutrophil depletion by CXCR2 or an anti-Ly6G antibody can restore neural function and reverse brain injury caused by hyperlipidemia, thus revealing the functional significance of neutrophils in the pathogenesis of ischemic brain injury (148). How neutrophils activated by the ischemic microenvironment affect brain remodeling and plasticity after stroke remains to be further elucidated. In conclusion, neutrophils play an important role in cerebral ischemic injury in hyperlipidemic mice. The results show that neutrophils can aggravate brain injury and lead to poor recovery of neurological function in a hyperlipidemia mouse model.

## The Value of Clinical Transformation

### Immunomodulatory Therapy Associated With Neutrophils

In rodent models, as in patients with ischemic stroke, leukocyte counts, cytokine levels and inflammatory marker levels are increased within hours after ischemia. This acute phase reaction is followed by immunosuppression, especially in stroke patients, which is characterized by decreased lymphocyte counts, decreased monocyte function, increased anti-inflammatory cytokine levels, lymphocyte apoptosis and spleen atrophy. These immune changes are associated with increased respiratory and urinary tract infections, and respiratory and urinary tract infections are the cause of the high incidence and mortality of stroke. Infection often occurs in patients with large stroke, low CD4 lymphocyte counts, and elevated levels of IL-10 and IL-6, which reflect immunodeficiency. How these systemic immune changes are mediated is not fully understood, but there is evidence that sympathetic activation is associated with the subsequent release of stress steroids and catecholamines (149). Therefore, cortisol and catecholamine levels are elevated in the most susceptible stroke patients, while the steroid antagonist and β-adrenergic receptor antagonist propanol can antagonize lymphocyte apoptosis and infection susceptibility after stroke in rodent models (1).

Peripheral blood neutrophils play an important role in predicting neurological deterioration and stroke prognosis. In patients, a higher peripheral blood neutrophil count and neutrophil-to-lymphocyte ratio (NLR) are independently associated with poor prognosis (150). The NLR can also predict poor prognosis and death in patients with AIS receiving intravascular therapy (151, 152). Neutrophils infiltrate diseased arteries at all stages of atherosclerosis, so inhibition of neutrophil recruitment is an important therapeutic strategy. Some recent studies also support our hypothesis. For example, compared with no treatment, long-term use of an anti-cathepsin G antibody in atherosclerotic mice was shown to reduce the size of atherosclerotic lesions (153). Studies have shown that CEACAM1 inhibits inflammation in reperfusion injury by controlling the MMP-9 level in neutrophils. Therefore, lower MMP-9 levels can reduce BBB breakdown, reduce secondary tissue damage, and improve neurological prognosis and survival after ischemic stroke (154) **(** **Table 1** **)**. Similarly, driving endogenous pathways to inhibit the activation of integrin induced by various chemokines may be an innovative therapeutic method for effectively inhibiting the adhesion of myeloid cells. For example, annexin A1 and growth differentiation factor 15 can antagonize the activation of β2 integrin induced by various chemokines, thus inhibiting neutrophil recruitment during chronic inflammation in mice (155). Studies have shown that pharmacological activation of the α-7 nicotinic acetylcholine receptor (α-7nAChR), an immunomodulatory receptor, during stroke promotes a decrease in the M1/M2 macrophage ratio (160, 161), thus conferring neuroprotection in rodent models. Similarly, in view of the different roles of different neutrophil phenotypes in ischemic stroke, activation of RXR-PPARγ can promote neutrophil polarization to the beneficial N2 phenotype, which may contribute to a reduction in inflammation; this explains the neuroprotective effect of the agonists besarotene and rosiglitazone (40, 158) **(** **Table 1** **)**.

**Table 1:** Immunomodulatory therapy associated with neutrophils.

Mechanism	Exemplar	Reference
Suppressing the generation or release of NETs and accelerating cleanup	PAD4 inhibitors and Externalized histone H4	(126, 134)
	DNase I digesting NETs	(156)
	Blocking ccl5-cxcl4 heterologous substance or blocking platelet neutrophil communication by P-selectin	(157)
Inducing differentiation direction of neutrophils	Activation of RXR PPARγ promotes neutrophil polarization to N2 phenotype	(40, 158)
Promoting vascular repairing	Silencing STING or an IFNAR antibody promotes angiogenesis and vascular repair	(59)
Inhibiting chemotactic factor	Annexin A1 and growth differentiation factor 15 antagonized chemokine-induced β2 integrin activation, thereby inhibiting neutrophil aggregation in chronic inflammation	(155)
Inhibition of blood brain barrier damage	CEACAM1 controls the secretion of matrix metalloproteinase-9 by neutrophils in inflammatory sites after brain-deficient stroke, thereby protecting the function of BBB and improving the outcome after stroke.	(159)

However, the mice used in existing experimental studies lack genetic and species diversity, and the differences between species make the generalization of the findings challenging. Therefore, further research is needed to overcome these obstacles.

Considering the importance of reticular structures in atherosclerotic formation, plaque instability, plaque erosion and atherosclerotic thrombosis, the pathway by which reticular structures are formed and their inflammatory responses are induced are obviously promising therapeutic targets in vascular inflammation after stroke. The interaction between neutrophils and platelets along blood vessels plays a decisive role in the release of NETs (162). Therefore, blocking platelet-derived ccl5-cxcl4 heterologous substances or blocking platelet neutrophil communication through high mobility group protein B1 or P-selectin may limit the release of NETs (112, 157). In a mouse model of atherosclerosis, therapeutic administration of Cl-amidine, a PAD4 inhibitor, prevents the release of nets and reduces vascular inflammation (134). It has a profound impact on the effects of chromatin NET degradation and DNaseI treatment on cardiovascular inflammation and thrombosis (156) **(** **Table 1** **)**. Moreover, NET chromatin is believed to activate absent in melanoma 2 (AIM2) inflammatory bodies in macrophages, thus promoting the production of IL-1β and IL-18 in atherosclerotic lesions in mice. The therapeutic inhibition of the AIM2 inflammasome in mice increases damage stability, so it may be an important therapeutic target downstream of the release of NETs (163). In conclusion, based on the results of studies of neutrophils in animal models, we expect more people to pay attention to the research progress in this field. We hope that future research can supplement the current knowledge and fill in related gaps. We also hope that animal studies can provide references for improving neurological function and long-term prognosis in patients with ischemic stroke through new treatment strategies.

## Conclusions

Although neutrophils have been neglected in the field of stroke for a long time, studies in the past decade have revealed the important regulatory function of neutrophils in the inflammatory process after stroke. According to these studies, these immune cells are important promoters at each stage of atherosclerosis, and the downstream process driven by neutrophil proteinases or neutrophils is an important new therapeutic target for improving the prognosis of stroke patients. However, neutrophils also have reparative effects on poststroke inflammation. Therefore, the two-sided nature of the effects mediated by neutrophil activity needs to be further explored. Whether these differences in the roles of neutrophils are the result of different functions of neutrophil subsets remains to be further confirmed. Therefore, regardless of the cause of these differences, we need to focus on the dual roles of neutrophils to identify neutrophil-targeted therapeutic strategies for poststroke inflammation.

## Author Contributions

RC wrote the initial draft. XZ contributed to reviewing the literature. The figures and submission were prepared by RC and XZ. LG, and XX prepared the final version. ZJ and XX recommended a structure for the review and substantially advanced the draft. All authors contributed to the article and approved the submitted version.

## Funding

This study was supported by the National Natural Science Foundation of China (No. 81870939 to XX), and the Natural Science Foundation of Hubei Province (No. 2020CFB613 to ZJ).

## Conflict of Interest

The authors declare that the research was conducted in the absence of any commercial or financial relationships that could be construed as a potential conflict of interest.

## Glossary

**Table:**

ROS	Reactive oxygen species
DAMPs	Damage-associated molecular patterns
CD	Cluster of differentiation
TCR	T cell receptor
Th cells	T helper cells
STAT	Signal transducer and activator of transcription
IFN	Interferon
IL	Interleukin
Treg cells	Regulatory T cells
NKT cells	Natural killer T cells
IRF	Interferon-regulatory factor
TGF-β	Transforming growth factor-&beta;
GM-CSF	Granulocyte-macrophage colony-stimulating factor
TNF	Tumor necrosis factor
AHR	Aryl hydrocarbon receptor
CCR	C-C chemokine receptor
APCs	Antigen-presenting cells
RA	Rheumatoid arthritis
NF-κB	Nuclear factor kappa-light-chain-enhancer of activated B cells
Tfh	Follicular helper T cells
CXCR	C-X-C chemokine receptor
CXCL	Chemokine (C-X-C motif) ligand
GCs	Germinal centers
LPS	Lipopolysaccharide
tMCAO	Transient middle cerebral artery occlusion
pMCAO	Permanent middle cerebral artery occlusion
CNS	Central nervous system
BBB	Blood-brain barrier
CCL	Chemokine (C-C motif) ligand
G-CSF	Granulocyte colony-stimulating factor
TLRs	Toll-like receptors
HMGB1	High-mobility group box 1
MAPCs	Multipotent adult progenitor cells
PLR	Platelet lymphocyte ratio
NLR	Neutrophil-to-lymphocyte ratio
VEGF	Vascular endothelial growth factor
FPR2	N-&ZeroWidthSpace;formyl peptide receptor 2
AIM2	Absent in melanoma 2
PAD4	Protein arginine deiminase 4
MPO	Myeloperoxidase
NETs	Neutrophil extracellular traps
ECM	Extracellular matrix
ICAM-1	Intercellular cell adhesion molecule-1
fMLF	N-formyl-methionyl-leucyl-phenylalanine
PAF	Platelet activating factor
PAMPs	Pathogen-associated molecular patterns
PRR	Pattern recognition receptors
NADPH	Reduced nicotinamide adenine dinucleotide phosphate
MMP-9	Matrix metalloproteinase-9
VLA-4	Very late antigen-4
ONC	Optic nerve crush
DRG	Dorsal root ganglion
NE	Neutrophil elastase
MIP-2	Macrophage inflammatory protein 2
CRAMP	DNA-cathelicidin-&ZeroWidthSpace;related antimicrobial peptide
NLRs	Nucleotide-binding oligomerization domain like receptors
NLRP3	NLR family pyrin domain containing 3
SDF-1	Stromal-derived factor 1
